# An Innovation of the Markov Probability Model for Predicting the Remaining Service Life of Civil Airport Rigid Pavements

**DOI:** 10.3390/ma15176082

**Published:** 2022-09-02

**Authors:** Baoli Wei, Chengchao Guo, Miaoyi Deng

**Affiliations:** 1 School of Civil Engineering and Architecture, Zhengzhou University of Aeronautics, Zhengzhou 450046, China; 2 School of Civil Engineering, Sun Yat-sen University, Zhuhai 519082, China

**Keywords:** airport runway, remain service life prediction, Markov process, survival analysis

## Abstract

In view of the time series update of airport runway health status detection data, the Markov chain of stochastic process theory was adopted. Considering the influence of aircraft traffic load, age, and pavement structure surface-layer thickness on the performance deterioration process of airport runways, the method of survival analysis was used. The parameter model of survival analysis was used to establish the duration function model of the four condition states of the airport runway PCI (pavement condition index). The Markov transition matrix for the performance prediction of airport runways was constructed. In order to evaluate the ability of the Markov transition matrix method to predict the trend of deterioration for PCI of the airport runway under different conditions of aircraft traffic volume and thickness of the runway pavement surface, a data set was constructed with the actual inspection data of the airport runway, and the corresponding samples were selected for analysis. The results showed that a Markov transition matrix for airport runway performance prediction, constructed based on survival analysis theory, can combine discontinuous inspection data or monitoring data with Weibull function survival curves. The method proposed in this paper can quantitatively predict the remaining service life of airport runways and provide support for cost-effective decisions about airport pavement maintenance and rehabilitation.

## 1. Introduction

The civil aviation industry is an important strategic industry for China’s economic and social development. The focus of the Outline of Action for the Construction of Four Types of Airports in China Civil Aviation (2020–2035) is to “Strengthen infrastructure operation monitoring and testing, improve the level of facility maintenance and management; build a smart airport, promote transformation and upgrading, and gradually promote the comprehensive IOT of all facilities, so that the state can be sensed and data can be obtained; realize the whole life cycle performance monitoring of major transportation infrastructure projects, with the help of big data, artificial intelligence and other innovative technologies, make full use of all kinds of resources, prediction and early warning, optimization control and other functions, and finally realize the intelligent operation of the airport”. It is clear that airport runway monitoring is very important for the intelligent operation of airports.

Historically, some airport sponsors have made decisions about pavement maintenance and rehabilitation (M&R) based on immediate need or experience rather than long-term planning or documented data on effective M&R methods. This approach does not allow the airport sponsors to evaluate the cost effectiveness of alternative M&R strategies, and it leads to the inefficient use of available M&R funds. To determine which M&R actions are preferable, one must be able to predict the future consequences of the various scenarios. This requires an understanding of the lifespan of the M&R method selected. Airports must also have a good understanding of the rate of pavement deterioration, with and without maintenance, and the causes of current pavement deterioration such as environmental or pavement-loading conditions. Predicting consequences of M&R scenarios requires experience and the application of best practices and engineering judgment in the decision-making process [1]. Remaining life predictions of airport pavements are important to airport pavement management. Remaining life predictions are necessary to develop optimum, multi-year M&R plans.

Therefore, this paper focused on the analysis of the method of remaining life prediction of airport runways. Based on the Markov process theory and combined with the survival analysis, the Markov transition matrix for airport runway performance prediction was constructed. Considering the effects of aircraft traffic volume, pavement surface thickness, and age, the Weibull-type survival function was constructed and the duration function model for different rating levels of airport runways was established. In this paper, a data set of a local civil airport runway testing was adopted and the Markov transition matrix method was used to predict and analyze the law of the airport pavement deterioration, aiming to provide a technical reference and support for maintenance decisions for airport pavement maintenance agencies.

## 2. Current Research Studies on the Prediction Approaches for the Remaining Life of the Airport Pavement

The pavement deterioration process [2] is a stochastic continuous change process due to data measurement errors, nonlinear behavior in the pavement deterioration process, and the role of unexplained influencing variables. The measurement of pavement deterioration and its modeling requires complex empirical models to capture it through certain probability distribution functions. The empirical models using traditional statistical regression techniques generally do not consider the stochastic nature of the pavement performance deterioration process and cannot explain the uncertainties in the deterioration process. In order to solve this problem, the probabilistic empirical model was developed. This kind of model mainly includes a Bayesian probability prediction model and a Markov probability prediction model, among others. The Markov model has a relatively wide range of practical applications.

In the 1980s, Golabi and Kulkarni et al. [3] jointly used the Markov prediction model and the linear programming method to develop and establish a network-level management system in Arizona. This was the first application of probabilistic prediction techniques and large-scale complex mathematical models in the field of pavement management. The application of Markov models on pavement condition prediction is based on two assumptions, namely, Markov inefficiency and a static transition process. The assumption of a static transition process is that the change in external factors will not affect the transition probability. That is, the probability of pavement performance deterioration remains unchanged, which is inconsistent with the actual deterioration process of pavement performance [4]. Since traffic loads and environmental conditions change throughout the analysis period during pavement service, the condition state transition probability of the pavement deterioration process is a time-varying random variable. The homogeneous Markov process assumes that the transition probability is a fixed value that does not change with time and cannot simulate the time-varying characteristics of the pavement performance deterioration. In recent years, researchers have begun to use the non-homogeneous Markov process to predict and analyze the pavement performance deterioration process. The non-homogeneous Markov process assumes that the transition probability is time-varying, which is more in line with the actual process of pavement performance deterioration. Hong et al. [5] considered the uncertainty of traffic volume and environmental behavior as well as the material properties and geometric variables of the pavement system. They developed a model for predicting time-varying flexible pavement deterioration processes based on non-simultaneous continuous Markov chains and explored the application of the model in pavement cost analysis. Abaza et al. [6] proposed an iterative linear stochastic pavement management model based on non-homogeneity.

However, it is worth noting that although the non-homogeneous Markov model truly simulates the random behavior of the pavement surface condition state changing with time, owing to the large amount of computation and the cumbersome process of model establishment, it is not yet widely used in airport pavement or road performance prediction in practice. In order to avoid the disadvantages of inhomogeneous and homogeneous Markov processes in practical applications, semi-Markov process prediction models have been developed. A semi-Markov process is a kind of synthesis of a homogeneous and a non-homogeneous Markov process. It not only considers the time phase of a homogeneous Markov process, but also takes into account the time-varying of a non-homogeneous Markov process in different time periods.

The semi-Markov model differs from the homogeneous and non-homogeneous Markov models. It estimates the probability of shifting the pavement condition by dividing the pavement life into uneven time intervals corresponding to the pavement performance curve (i.e., duration: the time for the pavement to completely shift to another condition state to remain in the current condition state). It assumes that the road surface condition states may remain of different durations before transitioning to other condition states, and estimates the transition probability for each duration by assuming that the durations follow a specific probability distribution. Nesbit et al. [7] analyzed the semi-Markov model in detail to predict the deterioration process of road pavement in a probabilistic way. They predicted the deterioration process of a heavy pavement performance in Manitoba by considering the probabilistic changes in pavement performance transition due to pavement maintenance and rehabilitation operations. Thomas et al. [8] proposed a semi-Markov model for pavement deterioration. This model assumes that the duration of each condition state obeys a Weibull distribution, so it is more flexible than the traditional Markov chain model. Based on the traditional Markov chain model and the proposed semi-Markov model, they performed Monte Carlo simulations of the deterioration of flexible pavements over time. The results showed that, in some cases, the semi-Markov model can better reflect the actual deterioration of flexible pavement than the Markov chain model.

Because the Markov process model requires a large number of samples to predict the transition probability of pavement performance, the analysis and calculation workload is large. To accommodate this situation, researchers in recent years have begun to combine econometric methods and Markov processes to predict pavement performance. Madanat et al. [9,10] and Yang et al. [11] pointed out that the use of econometric models to calculate the transition probability of the pavement surface performance predicted by the Markov process can reflect changes in road conditions on the basis of a large amount of historical data. The measurement economic methods can link the pavement deterioration process with relevant influencing factors using explanatory variables. In addition, this combined method provides more accurate predictions of pavement conditions than the uncombined method. The main econometric models used in the Markov model for predicting pavement performance include probabilistic models, logit models, and ordered probability models, among others. Yang et al. [12] compared the Markov process combined with the logit model prediction method and the artificial neural network model prediction method. They found that the two models have similar performance in single-year forecasting, but the Markov forecasting method combined with the logit models are more accurate than artificial neural networks in multi-year forecasts. Li [13] used an ordered probability model combined with a Markov model to obtain the transition probability for predicting the deterioration process of the PCI of pavement infrastructure. Yuan Jie et al. [14] incorporated the logit model into the Markov model and selected samples of measured PCI data from several airports in China to calculate the parameters in the Markov probabilistic prediction model and to predict the PCI deterioration process of the airport pavement. In addition to using economic methods to solve the problem of sample size, many scholars also use embedded optical fiber sensors in pavement structure to obtain sample data by on-site monitoring approaches. Imad Al-Qadi [15] installed 149 monitoring sensors on the flexible pavement of Cagliari-Elmas Airport in Italy to collect pavement performance information, providing data support for improving the design of airport pavement, evaluating runway performance, and predicting runway damage. Based on the runway monitoring system of Newark International Airport, Cook et al. [16] studied and analyzed the data results of the runway monitoring system of Newark International Airport, aiming to provide suggestions and support for the installation of the strain monitoring system of Honolulu Airport in 2016 and Kahului Airport in 2019. Braunfelds et al. [17] installed fiber Bragg grating (FBG) optical sensors on an asphalt concrete pavement in Latvia and collected and sorted out pavement performance data. They pointed out that temperature and strain data collected through on-site monitoring can provide data support for the evaluation and prediction of pavement structural life.

In addition, considering that the start time of pavement performance deterioration has significant uncertainty and randomness, the starting time data of condition change obtained from the pavement condition monitoring data have the characteristics of censored data [18]. For example, the pavement surface condition survey is generally carried out at certain intervals. When this survey to the pavement surface condition is in a certain condition state, it is not necessarily the start time of the deterioration of the pavement surface condition to the current condition state; it may be greater than the start time of the transition to the current condition state, which leads to the censoring of data [19]. In order to solve the problem of data censoring, some researchers combine survival analysis models and Markov processes to predict the condition state of an infrastructure. Mauch et al. [20] used the semi-parametric model of survival analysis combined with the Markov process to predict the performance deterioration process of concrete bridge decks using 10 years of inspection data in the Indiana bridge survey database. Khan et al. [21] introduced the survival analysis parametric model into the Markov process model to predict the road deterioration process caused by flooding. In addition, Scheidegger [22], Bai [23], and Kobayashi [24] applied survival analysis and Markov process combined models to predict the remaining life or life cost analysis of lighting facilities in urban pipelines, subways and highway tunnels, respectively.

From the above, it can be seen that due to the complexity of airport pavement remaining life prediction, many scholars have used different methods to predict the change law of pavement performance, or, based on the survey results of pavement performance, a statistical model that fits with it can be obtained. In general, in order to reflect the performance decay characteristics of different individual pavements under the influence of various observable factors and random factors in the development process of the pavement performance prediction model, both empirical and mechanistic–empirical prediction models are integrated with each other. The regression empirical prediction method introduces random factor variables to consider the influence of random components, and the Markov process model introduces statistical regression techniques or intelligent algorithms to take into account the utilization of data. For this reason, it is necessary to find a method to evaluate and predict the performance of airport pavement surfaces. The method is interpretable, takes into account the influence of different factors, and has a practical engineering value. The method is used to achieve a scientifically accurate prediction of the condition state of the airport pavement surface.

## 3. Research Methodology

**Figure 1:**
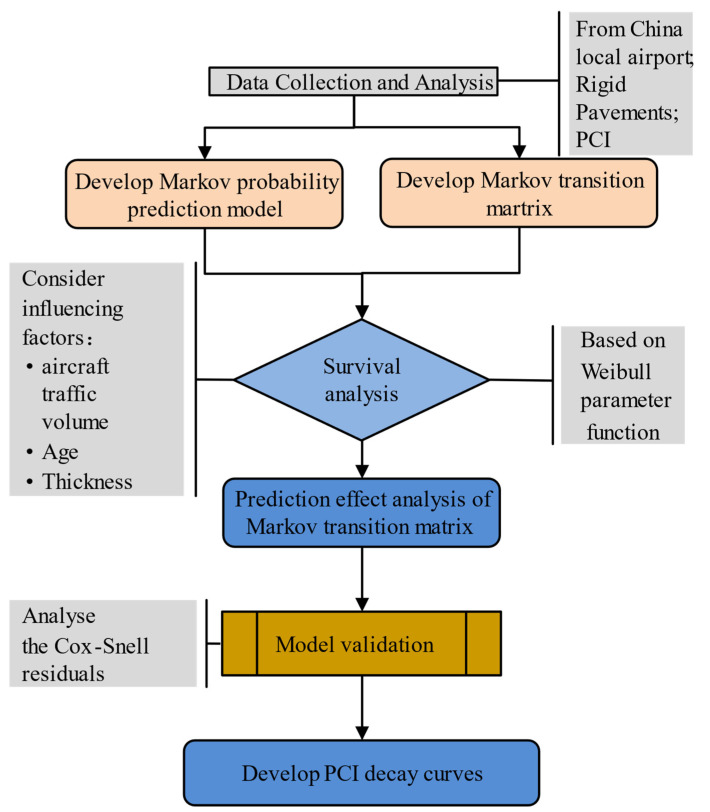
Research framework.

The Markov process is the theoretical basis of the widely used Markov prediction model of pavement performance, and the key to applying the Markov prediction model is to determine the transition probability matrix of the condition state. For the determination of the Markov state transition probability in the process of pavement performance deterioration, if *p_ij_* is used to denote the probability of transitioning state *i* to state *j*, the traditional method generally assumes that the transition of pavement service performance from a low-level state to a high-level state will not occur under normal maintenance conditions. That is, *p_ij_* = 0 when *i* > *j*. Because its service performance level will not decrease too quickly within one year, it is considered that the pavement service performance decline only occurs between two levels. That is, when *j − i* ≥ 2, *p_ij_* = 0. In fact, according to the length of the maintenance cycle, the pavement service performance level may also decrease by two, three, or more levels within one year. That is, when *j − i* ≥ 2, *p_ij_* ≠ 0. In this case, the transition probability matrix reflects inhomogeneity. In this section, in view of the deficiencies of traditional methods to establish a transition probability matrix, the survival analysis method is applied, and the non-homogeneous Markov state transition probability matrix is established by considering the influence of multiple factors. The construction method of the transition probability matrix in airport runway performance prediction was studied. The research framework of this paper is shown in Figure 1.

### 3.1. Construction of Markov Transition Matrix

During the deterioration of airport pavement performance, the stochastic process satisfies the Markov property if the future conditions of the airport pavement depend on the current conditions of the pavement rather than on past conditions. Although the pavement performance decay process has random characteristics, the state and time in the pavement performance decay process are both continuous variables. Therefore, the following provisions are often made when applying the Markov prediction model [25]:

1. Although the deterioration process of pavement performance is continuous in time, the test of pavement performance status and the investigation and inspection of pavement damage are carried out within a fixed period. Therefore, the time can be discretized into a period of one year or a period of five years for a detection cycle.
2. The value of pavement condition index is generally continuously distributed in a certain range, such that PCI takes the value of 0~100. However, in order to facilitate comparison, analysis, and grasp of the pavement performance status, a fixed interval method is usually used to discretize the state value range into a limited evaluation standard level.
3. The median value of the state interval can be regarded as the state representative value, and the state distribution probability can be regarded as the weight, and the two are multiplied to obtain the determined value of pavement performance prediction.

Based on the above points, the Markov model prediction method of pavement performance is as follows:

1. Select the predictor index and divide the state space of the index. The pavement surface performance prediction uses the pavement condition index PCI (0 to 100) as a predictive index. In order to better reflect the deterioration law of the airport pavement performance, it is necessary to discrete the PCI of airport pavement into various state spaces. According to the characteristics of the airport pavement PCI, the grades are divided into 5 grades, namely, good, satisfactory, fair, poor, and very poor. The state space indicator variables are 1~5, respectively. It is assumed that the probability distribution of the performance status of the pavement in various states at any time *t is X*(*t*), and the observation period of the probability transition is in years. The initial moment of the process is recorded as *t*_0_, and then the initial distribution vector of pavement performance is $X\left(t_{0}\right)=\left(a_{01},a_{02},\cdots ,a_{05}\right),\;\sum_{i}a_{0i}=1$.
2. Establish the Markov transition probability matrix. The Markov chain mainly predicts the deterioration process of pavement performance with time through the transition probability matrix. If it is assumed that routine maintenance is performed, the pavement performance will not gradually transition from the original low-level state to a high-level state. That is, if *i* > *j*, then *p_ij_* = 0. This performance level will not be improved to a higher level without considering pavement maintenance, and it can be determined that this performance deterioration is only carried out to the lower level. Considering the inhomogeneity of the transition probability, the transition probability matrix is as Equation (1), that is, the Markov transition matrix:
  (1) $$P=\left[\begin{array}{ccccc} p_{11} & p_{12} & p_{13} & p_{14} & p_{15}\\ 0 & p_{22} & p_{23} & p_{24} & p_{25}\\ 0 & 0 & p_{33} & p_{34} & p_{35}\\ 0 & 0 & 0 & p_{44} & p_{45}\\ 0 & 0 & 0 & 0 & p_{55} \end{array}\right]$$
3. In order to facilitate the calculation during prediction, in general, it is assumed that no transition occurs in the performance state of the pavement. Based on the Chapman–Kolmogorov equation [26], it can be known that, with the transition matrix *P^t^* from the state at time *t*_0_ to the state at any time *t,* according to the probability vector *X*(0) of the initial state, the Markov prediction model can be written as Equation (2) below:
  (2) $$X\left(t\right)=X\left(0\right)P^{t}$$
  Equation (2) can be used to predict the performance of the pavement in each state at any time in the future.

### 3.2. Method of Establishing Transition Matrix Based on Survival Analysis

Based on the construction and analysis of the Markov transition matrix, when estimating the parameter model of the pavement performance deterioration process, the corresponding discrete-time, state-limited Markov model transition probability can be calculated. That is, the transition probabilities of discrete-time, state-limited processes are determined from time-continuous Markov processes. In the two-state case, the transition probability of state *i* is$\;R\left(t,\Delta \right)$, and survival analysis theory [27] is used to obtain its expression as follows.

(3) $$R\left(t,\Delta \right)=\frac{Prob\left(t<T<t+\Delta \right)}{Prob\left(T>t\right)}=\frac{F\left(t+\Delta \right)-F\left(t\right)}{1-F\left(t\right)}=\frac{F\left(t+\Delta \right)-F\left(t\right)}{S\left(t\right)}$$

where *F*(*t*) is the cumulative distribution function of the random variable duration t; *S*(*t*) is the survival function. Therefore, if *F*(*t*) is known, *R*(*t*,Δ) can be calculated for arbitrary values of *t* and Δ. That is, given a stochastic process model describing a probability density function of duration *t*, the transition probability of the corresponding discrete state stochastic process can be determined from *R*(*t*,Δ).

For infrastructure performance deterioration processes, when a probability density function of the performance deterioration process is used and a set of influencing factors (or explanatory variables) is used to analyze the performance deterioration process of a runway, the hazard rate function can be based on survival analysis theory, using the Weibull probability density function. The hazard rate function of the Weibull probability density function is as follows:

(4) $$\lambda \left(t\right)=\lambda p{\left(\lambda t\right)}^{p-1}=p\lambda^{p}t^{p-1}$$

where *λ* and *p* are parameters to be estimated in Equation (4). Since the expectation $E\left[T\right]=\lambda^{-p}$ of the Weibull probability density function of the duration *T* of a state, depending on the value of *p*, the hazard rate function can respond to negative time dependence or time irrelevance (independence). Based on the survival analysis method, the survival function *S*(*t*) can be calculated using the following Equation (5):

(5) $$S\left(t\right)=e^{-\Lambda \left(t\right)}$$

where $\Lambda \left(t\right)={\left(\lambda t\right)}^{p}$, according to Equation (3), and the expression of the survival function *S*(*t*) can be obtained as follows:

(6) $$S\left(t\right)=e^{-{\left(\lambda t\right)}^{p}}=\exp[-\left(\lambda t{)}^{p}\right]$$

Therefore, the probability *P_ii_* of maintaining state *i* can be calculated using Equation (7):

(7) $$P_{ii}=1-R\left(t,\Delta \right)=\frac{\exp\left[-\lambda^{p}{\left(t+\Delta \right)}^{p}\right]}{\exp\left[-{\left(\lambda t\right)}^{p}\right]}$$

The probability of transitioning from state *i* to state *j*, *P_ij_*, can be obtained as follows:

(8) $$P_{ij}=1-P_{ii}=R\left(t,\Delta \right)=1-\frac{\exp\left[-\lambda^{p}{\left(t+\Delta \right)}^{p}\right]}{\exp\left[-{\left(\lambda t\right)}^{p}\right]}$$

From Equations (6)–(8), *λ* is determined by Equation (3); that is, if the hazard rate function *λ*(*t*) of Weibull distribution is determined, the probabilities *P_ii_* and *P_ij_* for any moment *t* and time interval Δ can be calculated using Equations (7) and (8).

## 4. Engineering Application Case Analysis and Results

### 4.1. Project Overview and Data Preparation

**Figure 2:**
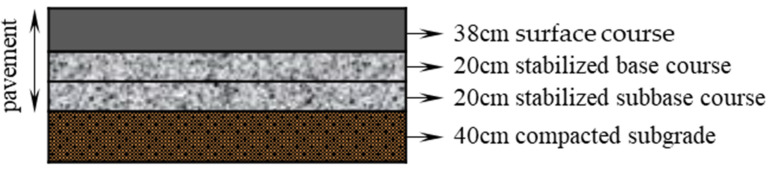
Structure of the airport rigid pavement.

In order to study the application and effect of the Markov transition matrix method in pavement performance prediction, when analyzing the trend of pavement condition grade deterioration, the detection data of a local civil airport (called the A airport in this paper) were used as the data source. This airport is the Chinese trunk transport airport and a national first-class aviation port. The south runway of this airport is 3400 m long and 45 m wide. The structural layer of the airport runway consists of a 38 cm-thick C40 cement concrete surface layer, a 40 cm-thick lime and fly ash stabilized gravel base course, and a 40 cm-thick compacted soil subgrade. A cross-section of the airport runway structure is shown in Figure 2.

To avoid new errors during data preprocessing, the data were preprocessed according to the data processing method in the literature [28]. Based on the annual number of landings and takeoffs of major aircraft types and the composition of the pavement surface structure, the sample individuals of the A airport pavement were grouped. Excluding some of the more specific or doubtful individual data points, 91 groups of rigid pavement data were obtained.

When predicting pavement condition, the numerical value of PCI was used to distinguish different state levels of the pavement performance deterioration process. The PCI is a numerical indicator that rates the surface condition of the pavement [29]. The PCI provides a measure of the present condition of the pavement based on the distress observed on the surface of the pavement, which also indicates the structural integrity and surface operational condition (localized roughness and safety). The specific state levels were divided according to the division method in the literature [30,31], as shown in Table 1.

In the survival analysis of the airport pavement performance deterioration process, the dependent variable is the time that the pavement maintains a certain state under given conditions. This is the duration which is represented by TIS (time in state) in this paper. TIS represents the time spent by the pavement sample in a specific state *i* of deterioration (i.e., the sojourn time). The approximation method proposed by Sobanjo [32] was used to extract the TIS from the observed condition rating over time. The method is as follows.

1. When a condition state of an individual pavement drops by one step between two consecutive inspections, the actual transition time is approximated to occur exactly in the middle of the two inspection times.
2. When a condition state of an individual pavement drops by two steps between two consecutive inspections, the TIS of the intermediate state is approximately half the inspection period.

The occurrence of left-censored observations was eliminated based on the second approximation above so that the observed data were all right-censored or failure data (i.e., exact life data). Since the data used in this paper cover a time span of 20 years, most of the TIS observations extracted from the dataset were right-censored. In addition, age was defined as the starting point from the latest pavement overhaul or when it was completed and put into use until it degenerates to the current state. In order to facilitate the analysis, this paper used the years at the detection time to approximate the age of the right-censored pavement individuals as the years of use for entering the current condition state. According to the above method, the finally obtained survival analysis data are shown in Table 2 (only some samples are shown).

### 4.2. Estimation Results of Survival Analysis Parametric Model

Using the above data set, the duration models for state 1 and state 2 were estimated using Equation (4), based on a Weibull distributed survival time function of the runway pavement performance deterioration process containing the main influencing factors (explanatory variables). 

The main influencing factors consider the aircraft traffic volume, pavement thickness, and age, in which the aircraft traffic volume adopts the natural logarithm value of the average annual take-off and landing sorties of the main aircraft operating at the airport when the current state is transitioned. The Weibull parameter function expression of the hazard rate is as follows:

(9) $$\left\{\begin{array}{l} \lambda \left(t\right)=p\lambda^{p}t^{p-1}\\ ln\lambda =\beta_{1}+\beta_{2}lgN+\beta_{3}H+\beta_{4}Age \end{array}\right.$$

In Equation (9), *β_i_* is the coefficient to be estimated for the influencing factor. The other parameters are as described in the previous section. The duration function model of state 1 of the PCI assessment level of the A airport pavement was estimated, and the specific parameter estimation results are shown in Table 3.

From the results of parameter estimation in Table 3, it can be concluded that the significance level of coefficient estimates for all the influencing factors was less than 0.1, except for the age variable, which was not statistically significant. This situation indicates that the influencing factors considered have a significant impact on the model prediction effect. The estimated value of the coefficient of the aircraft traffic volume lgN was −0.18, that is, the aircraft traffic volume and the hazard rate were negatively correlated, which means that with the increase in the aircraft traffic volume, the hazard rate gradually increased. Moreover, as the frequency of the aircraft load acting on the airport pavement increased, the probability of the airport pavement maintaining the current state level gradually decreased; furthermore, the greater the risk that the TIS will maintain its current state level for the duration of the airport pavement, the greater the probability that it will transition to the next state level.

In addition, the estimated value of the coefficient of the pavement thickness H was 0.02. This indicates that the pavement thickness and the hazard rate were positively correlated. That is, with an increase in the pavement thickness, the hazard rate gradually decreased. As the thickness of the airport runway pavement increased, the structural bearing capacity of the airport runway increased. The probability of the airport pavement maintaining the current state level also gradually increased. Therefore, the smaller the risk that TIS will maintain the current state level for the duration of the airport runway, the smaller the probability of its transition to the next state level. 

In addition to the influence of the above-mentioned explanatory variables, the value of the parameter *p* was estimated to be 8.49, and the 95% confidence interval was 6.24 to 11.57. This indicates that *p* was significantly different from 1 at the 5% level of significance. Because *p* > 1, the hazard function *λ*(*t*) in the form of a Weibull probability distribution was an increasing function of *t*, reflecting a positive temporal correlation. Secondly, the estimated value of the coefficient *β*_4_ of age was not statistically significant. This indicates that the duration of the deterioration process of airport pavement performance at the state 1 level was not related to age. That is, it has a non-aftereffect property.

### 4.3. Prediction Effect Analysis of Markov Transition Matrix

Since the state duration of the airport pavement deterioration process has obvious Markov characteristics, this paper used the Markov model to analyze the stochastic process of the airport pavement deterioration. The Markov transition matrix of the pavement performance deterioration process was established. The following is an analysis of the prediction effect and prediction performance of the Markov transition matrix.

#### 4.3.1. Evaluation of the Model Fitting Effect

The graphical method or residual analysis method can be used to evaluate the effect of the survival analysis model fitting survival function. For the residual analysis of the survival analysis model, many researchers have pointed out a variety of residual analyses; however, the most effective one is the Cox−Snell residual [33]. Its value is the estimated value of survival probability at time *t* obtained using the fitting model for individual survival time *t_i_* under the influence of covariate *x_i_*. It can be represented with Equation (10).

(10) $$CS_{j}=-\log\hat{S_{j}}\left(t_{j}\right)$$

In the formula, $\hat{S_{j}}\left(t_{j}\right)$ is an estimate of the probability of survival at time *t* based on the fitted model. Cox and Snell determined that, if the model can fit the actual data well, then the residual error $CS_{j}$ approximately follows an exponential distribution function with mean 1. Therefore, the curve of the relationship between the cumulative hazard rate of $CS_{j}$ and the residual error $CS_{j}$ itself should be a straight line with slope 1. In addition, it can be seen from Equation (9) that the Cox–Snell residual error cannot be negative: the estimate survival probability is (0,1), so the residual error$\;CS_{j}$ is not a symmetric distribution about 0.

**Figure 3:**
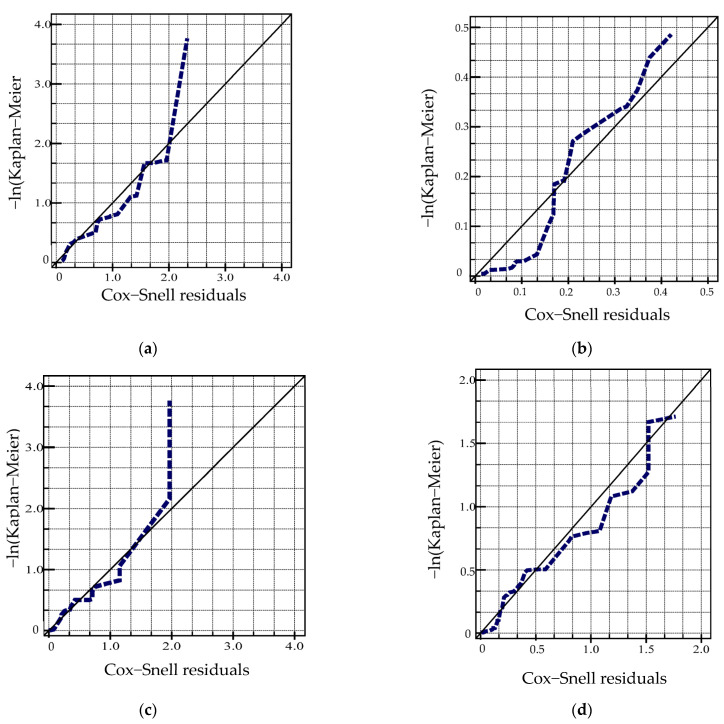
Residuals for the model of state duration. (**a**) State 1; (**b**) State 2; (**c**) State 3; (**d**) State 4.

The Cox–Snell residuals of the Weibull parameter models for the duration model of different states of the airport pavement performance deterioration are shown in Figure 3. It can be shown in Figure 1 that the residuals of the duration model for each state level only deviated from the larger value of the Cox−Snell residual error, and it is a normal situation for data sets with more data to have outliers. Therefore, the fitting effect of the Weibull parameter model of four state (1,2,3,4) durations was good. The $CS_{j}$ can be used to analyze the prediction effect of the Markov transition matrix.

**Figure 4:**
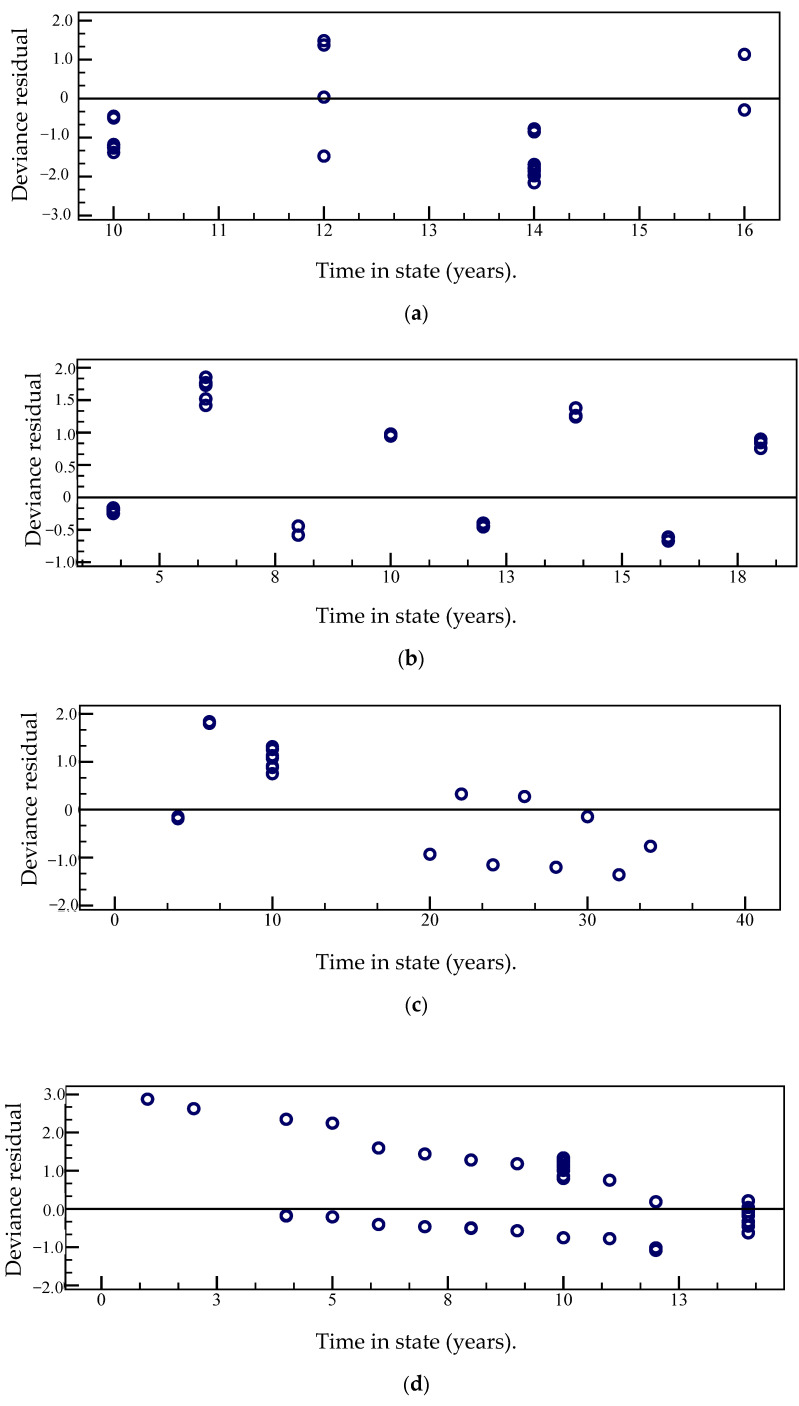
Deviation of the fit model for the sojourn time of four states. (**a**) State 1; (**b**) State 2; (**c**) State 3; (**d**) State 4.

Another way to evaluate model fit is to use bias. If the model fits the actual data well, these residuals are symmetric residuals about 0, and based on these residuals, it is easier to check for outlier data values. The deviations in the Weibull parameter models for different state levels and durations of the airport pavement PCI deterioration models were made as shown in Figure 4.

It is shown in Figure 4 that, with the exception that the deviation of state 4 occurred in relatively few extreme cases at the initial stage of the observation duration, the deviation of the duration observation value of other state levels was basically symmetrically distributed around the zero value. This verifies the correctness of the airport pavement PCI deterioration model established in this paper.

#### 4.3.2. Evaluation for Model Prediction Performance

Using the survival analysis method, based on the Markov stochastic process theory, the transition probability matrix *P_ij_*(*t*) of any state of the airport pavement deterioration process at any time can be obtained. The transition matrix was constructed and used to predict the airport pavement deterioration process at any time *t*. The specific prediction steps are as follows:

The initial time of the pavement deterioration process is recorded as t0, then the initial distribution vector of pavement performance is *COND*^T^(*t*_0_) = (*a*_01_,*a*_02_,…, *a*_05_), ∑*a*_0*i*_ = 1, where *a*_0*i*_ is the probability that the airport pavement is in state *i* at time *t*_0_, *i* = 1,2,… 5. Then, the transition probability matrix *P* from the state at time *t*_0_ to any state at time *t* can be obtained from the Chapman–Kolmogorov equation, that is, the product of the initial distribution vector *COND*^T^(*t*_0_) and the predicted transition probability matrix *P_ij_*(*t*):$COND^{T}\left(t\right)=COND^{T}\left(t_{0}\right)P_{ij}\left(t\right)$. According to the PCI value of the state evaluation grade vector PCI^T^ = (100~85, 85~70, 70~55, 55~40, 40~0) to determine the airport pavement after time *t*, the PCI predicted value is ${\mathrm{PCI}}_{t}=COND^{T}\left(t\right)\times \mathrm{PCI}$. Conversely, the state level vector *STR*^T^ = (5, 4, 3, 2, 1) can be used to determine the state level prediction value of the airport pavement after time *t* $STR_{t}=COND^{T}\left(t\right)\times STR$.

**Figure 5:**
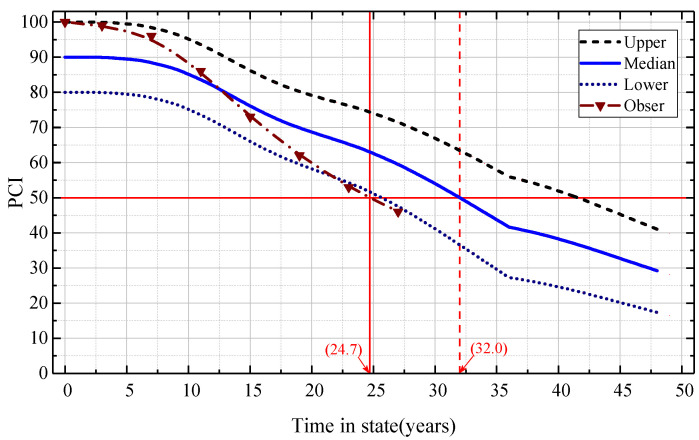
PCI prediction of the Markov model for airport pavement sample Data1S08.

**Figure 6:**
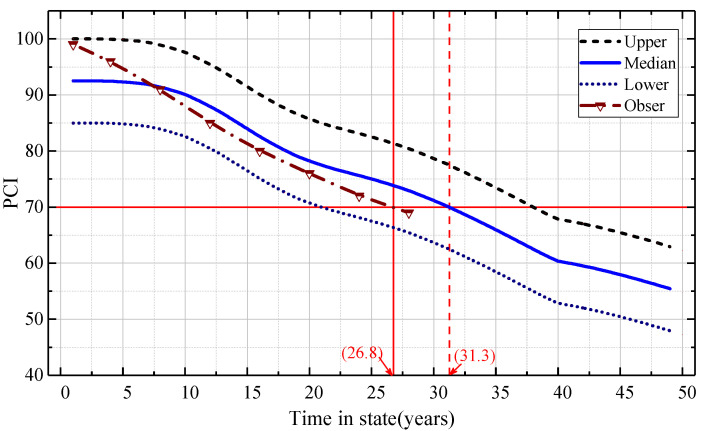
PCI prediction of the Markov model for airport pavement sample Data2S22.

If the initial state vector $COND^{T}\left(t_{0}\right)=(1\;0\;0\;0\;0)$, taking the 8th sample data point and the 22nd sample data point of the data set above as the analysis cases, respectively, the Markov transition matrix was used to predict the PCI attenuation curve and the state level transition curve of the airport pavement. The performance prediction results of the two sample pavements are shown in Figure 5 and Figure 6. The blue solid line in the figure represents the median prediction curve of the PCI value status interval of the airport pavement. The upper and lower dashed lines are the upper and lower PCI prediction curves, respectively. The dotted line with the triangle mark is the measured PCI value of the airport pavement.

It is shown in Figure 5 and Figure 6 that the measured PCI data were basically distributed within the prediction interval of the model prediction curve. This shows that the Markov transition matrix can predict the PCI data of two samples (8th and 22nd) well.

In addition, it can be concluded from Figure 5 that, in the curve of the deterioration process predicted by the Markov transition matrix, the upper limit of the prediction interval was close to the measured value in the early stage of the deterioration process, while the median value of the prediction interval was close to the measured value in the middle and late stage of the deterioration process. This indicates that the Markov transition matrix could predict the duration (or use time) of states 3–5 (fair to poor) better than that of state 1 (good) or state 2 (satisfactory). The main reason may be related to the mechanical characteristics of pavement performance deterioration. In the early stage of pavement service, due to the short service time and the small number of aircraft loads, the structural bearing capacity of pavement was basically maintained at the design level, and the probability of maintenance at the good level was relatively large. Thus, the PCI value was basically maintained at the high level. However, in the middle and late stage of pavement service, due to the increase in service time and the accumulation of aircraft load times, the structural bearing capacity of the pavement was gradually consumed. The probability of the grade transition of the pavement performance condition increased, so its PCI value was closer to the medium and low level.

#### 4.3.3. Pavement Prediction Performance for Different Aircraft Traffic Volumes

**Figure 7:**
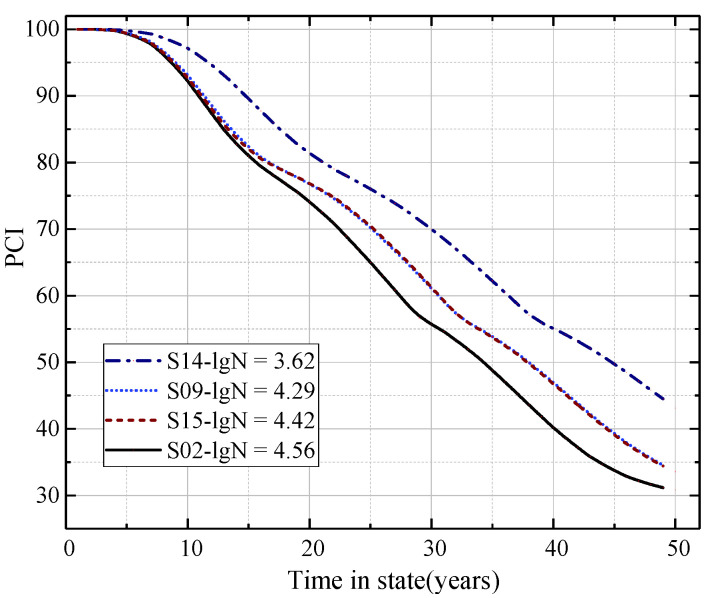
Prediction of PCI for the model with a different aircraft traffic volume (H = 32 cm).

In order to analyze the influence of aircraft traffic volume on the prediction performance of the Markov transition matrix, the pavement samples with the same pavement thickness but different aircraft traffic volumes were selected for analysis. Specifically, sample S14 (lgN = 3.62) and sample S9 (lgN = 4.29), sample S15 (lgN = 4.42), sample S2 (lgN = 4.56), all had individual pavement concrete surface thicknesses of 32 cm. The PCI prediction curve was constructed as shown in Figure 7.

It can be seen from Figure 7 that the Markov transition matrix was able to predict obvious differences in the decay trend of pavement PCI for different aircraft traffic volumes. As the aircraft traffic volume increased, TIS decreased. The higher the aircraft traffic volume, the greater the decay rate of the PCI. This is consistent with the discussion conclusion of the model parameters in Section 4.2 above. This indicates that with the increase in aircraft traffic volume, the damage degree of the pavement increases. As a result, the TIS (or service time at the same state) decreases. This also proves that the Markov transition matrix can better predict the decay trend of PCI for individual pavements with different aircraft traffic volumes (limited to the range of aircraft traffic volume contained in the data set).

It is worth noting in Figure 7 that the slope of the decay curve of PCI for the pavement with the aircraft traffic volume lgN = 3.62 was significantly different from the slope of the PCI decay curve of other aircraft traffic volumes. This indicates that, at the initial stage of the airport pavement service, due to the structural or material characteristics of the airport pavement, the increase in aircraft traffic volume had little impact on pavement damage. However, when the aircraft traffic volume of airport pavement increased to a certain value, the increase in aircraft traffic volume had a significant impact on pavement damage. This also indirectly shows that the aircraft load has a certain cumulative effect on the pavement damage.

In addition, it is worth noting that the prediction curves of aircraft traffic volume lgN = 4.29 and lgN = 4.42 basically coincided. This shows that the decay trend of PCI at these two aircraft traffic volume levels was basically the same. However, the prediction curves of aircraft traffic volume lgN = 4.42 and lgN = 4.56 were significantly different, which indicates that the decay trend of PCI at these two aircraft traffic volume levels was significantly different.

The main reason may be that the logarithm of the aircraft traffic volume was converted in this paper for analysis. Therefore, the logarithm difference of the three levels of aircraft traffic volume was small (4.42–4.29 = 0.13, 4.56–4.42 = 0.14). In fact, the difference in the three levels of actual traffic volume was about 6804 sorties, which is quite different. The possible reason is that when the thickness of the cement concrete surface of the airport pavement is 32 cm and the number of aircraft traffic volume reaches a certain level, the performance deterioration trend of the airport pavement will suddenly change. In other words, for a certain grade level of airport, there is an extreme point of its aircraft traffic volume. When the value of aircraft traffic volume exceeds this extreme point, it may cause significant damage to the airport pavement.

#### 4.3.4. Pavement Prediction Performance for Different Pavement Thicknesses

**Figure 8:**
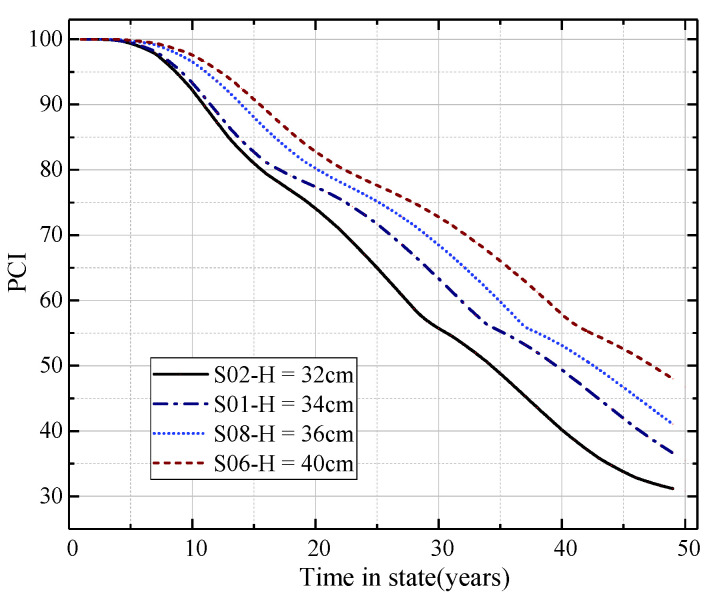
Prediction of PCI for the model with different pavement thicknesses.

In order to analyze the influence of pavement thickness on the prediction performance of the Markov transition matrix, samples S01 (lgN = 4.56, H = 34 cm), S02 (lgN = 4.56, H = 32 cm), S06 (lgN = 4.65, H = 40 cm), and S08 (lgN = 4.65, H = 36 cm) with different pavement thickness values at similar aircraft traffic volumes were selected from the data set. The PCI prediction curves are shown in Figure 8.

From Figure 8, it can be seen that the Markov transition matrix prediction curve can better predict the PCI of pavements with different surface thicknesses (under the condition of constant aircraft traffic volume). The PCI decay curves of different pavement thicknesses were obviously different. As the thickness of the pavement decreased, the value of the PCI decreased. The smaller the pavement thickness, the greater the decay rate of the pavement PCI. This is consistent with the analysis conclusions of the model parameters in the previous section. It shows that, with the decrease in the pavement thickness, the load-bearing capacity of the pavement structure decreases. As a result, the greater the damage degree of the pavement under the same aircraft traffic volume, the greater the effect on the reduction in the PCI. Thus, greater damage to the pavement under the same aircraft traffic volume level affects the PCI value reduction. This also proves that the Markov transition matrix can better predict the PCI decay trend for pavement individuals with different pavement thicknesses (only limited to the pavement thickness range contained in the data set).

## 5. Conclusions

According to the complex, random, dynamic characteristics of airport pavement performance deterioration, the transition matrix of the Markov process model of airport pavement performance deterioration was constructed using the Weibull function parameter model of survival analysis, considering the influence of pavement thickness, age, and aircraft traffic volume, based on pavement condition test data. The main conclusions of this paper are as follows.

1. With the increase in the duration of maintaining a certain state, the hazard rate of the airport pavement maintaining a certain state increased monotonically, reflecting a positive correlation with time. The semi-Markov property of the airport pavement performance deterioration process model was verified from the model parameter estimation results. This shows that the time distribution function has obvious characteristics of Weibull function distribution. The hazard rate function *λ*(*t*) was an increasing function of *t*, and the deterioration process of the airport pavement condition index was related to the state duration. This also shows that the probability density distribution of the random variable TIS of the deterioration process is not only dependent on the current state, but also related to the next state.
2. The fitting effect of the model was verified from the Cox–Snell residual error graph. The residuals of the state duration model at each state only had some deviations at the larger values of the Cox–Snell residuals. This shows that the four states’ (1,2,3,4) duration parameter model fitting effects were better. The model fitting effect was further evaluated from the deviation graphs of the state duration parameter models of different states of the airport pavement performance deterioration model. With the exception that the deviation of state 4 occurred in relatively few extreme cases at the initial stage of the observation duration, the deviation of the duration observation value of other state levels was basically symmetrically distributed around the zero value. This verifies the correctness of the airport pavement PCI deterioration model established in this paper.
3. The effect of predicting the PCI decay curve using the Markov transition matrix was analyzed by randomly selecting individual samples of airport pavement data sets. The Markov transition matrix could better predict the PCI of the individual samples. The generalization performance of airport pavement PCI prediction based on the Markov transition matrix was analyzed using data outside the data set. The results showed that the Markov transition matrix method was more sensitive to the pavement thickness than the aircraft traffic volume. The marginal effect of pavement thickness was significantly greater than that of aircraft traffic volume. These characteristics show that the Markov transition matrix based on a certain number of PCI data sets can better predict the attenuation trend of PCI for other similar airport pavements.

## Figures and Tables

**Table 1:** Evaluation criteria for the apparent condition of the airport’s rigid pavement surface.

Rating	Good (1)	Satisfactory (2)	Fair (3)	Poor (4)	Very Poor (5)
PCI	PCI ≥ 85	70 ≤ PCI < 85	55 ≤ PCI < 70	40 ≤ PCI < 55	PCI < 40

**Table 2:** Partial data source of airport pavement.

Group ID	Data Set	Time-in-State of Good State	Censor or Not	Age (Year)	Average Annual Aircraft Traffic	Thickness (cm)
ID	DS	TIS-1S	Censor	Age-1S	N-1S	H
1	1	10	1	8	35,934	34
2	1	10	0	8	35,934	32
20	2	12	0	12	74,325	38
21	2	10	1	8	74,325	38
33	2	14	1	12	74,325	34
34	2	12	0	12	74,325	34

Note: 1. Censor or not, where 1 means right-censoring, and 0 means state transition occurs. 2. Age (age-1S) is the time from the last time the pavement was overhauled or completed and put into use until it degenerated to the current state. 3. Average annual aircraft traffic is the average annual take-off and landing sorties of the main aircraft operating at the airport when the current state is transitioned. 4. Thickness is the thickness of the cement concrete surface of the airport pavement, in cm.

**Table 3:** Weibull function parameter estimates of State 1 TIS.

Coefficient	Estimated Value	Standard Deviation	Wald Chi-Square Value	*p* Value	Attributes
*β*_1_	2.93	0.32	81.47	<0.0001	intercept
*β*_2_	−0.18	0.08	4.90	0.03	slope
*β*_3_	0.02	0.01	4.19	0.04	slope
*β*_4_	−0.0086	0.02	0.24	0.62	slope
function parameter	estimated value	standard deviation	95% confidence interval		attributes
1/*p*	0.12	0.02	0.09	0.16	scale parameter
*p*	8.49	1.34	6.24	11.57	shape parameter
Model fit statistics					
−2 log likelihood 43.677					
AICC (smaller is better) 54.271					
BIC (smaller is better) 67.041					

## Data Availability

The figure and table data used to support the findings of this study are included in the article. In addition, the data and the models of analysis are available from the corresponding author upon request.
